# Exploring and expanding the chemical multiverse of peptides

**DOI:** 10.1039/d5sc04465k

**Published:** 2025-12-17

**Authors:** Edgar López-López, Jean Paul Sánchez-Castañeda, Massyel S. Martinez-Cortés, Cesar de la Fuente-Nunez, José L. Medina-Franco

**Affiliations:** a DIFACQUIM Research Group, Department of Pharmacy, School of Chemistry, Universidad Nacional Autónoma de México Avenida Universidad 3000 Mexico City 04510 Mexico medinajl@unam.mx jeanpaul.sac@comunidad.unam.mx mass.bqd@gmail.com jose.medina.franco@gmail.com; b Department of Chemistry and Graduate Program in Pharmacology, Center for Research and Advanced Studies of the National Polytechnic Institute Section 14-740 07000 Mexico City Mexico elopez.lopez@cinvestav.mx edgar.lopez.593@hotmail.com; c Machine Biology Group, Departments of Psychiatry and Microbiology, Institute for Biomedical Informatics, Institute for Translational Medicine and Therapeutics, Perelman School of Medicine, University of Pennsylvania Philadelphia Pennsylvania USA cfuente@upenn.edu; d Departments of Bioengineering and Chemical and Biomolecular Engineering, School of Engineering and Applied Science, University of Pennsylvania Philadelphia Pennsylvania USA; e Department of Chemistry, School of Arts and Sciences, University of Pennsylvania Philadelphia Pennsylvania USA; f Penn Institute for Computational Science, University of Pennsylvania Philadelphia Pennsylvania USA

## Abstract

Peptides occupy a unique and rapidly expanding domain within the broader chemical space, offering exciting opportunities for therapeutic, nutritional, cosmetic, and materials applications. While efforts to characterize chemical space have traditionally focused on small molecules, growing evidence underscores the value of extending this concept to include peptide-based compounds. In this review, we survey recent advances in the exploration and expansion of the peptide chemical space, focusing on short peptides that bridge chemoinformatics and bioinformatics perspectives. We begin by briefly discussing the impact and applications of peptides in various research and industry areas and then examining the theoretical and practical size of the peptide chemical space, emphasizing how naturally occurring and synthetic peptides vastly increase its diversity. We then discuss molecular representations—from conventional notations to specialized peptide descriptors—highlighting their impact on library design, structural analyses, and activity predictions. Visualization methods and machine learning models are presented as tools for mapping structure–property and structure–function relationships. Next, we explore computational strategies for *de novo* peptide generation, driven by advances in generative modeling and high-throughput screening. Throughout, we emphasize the role of open-source resources and integrated computational pipelines that combine chemo- and bioinformatics approaches to enhance data quality and predictive performance. We conclude by identifying major challenges—such as the complex structural landscape of peptides, data curation, and the need for consensus screening methods—and outline emerging opportunities for further expanding and refining the peptide chemical space.

## Abstract 2

Peptides occupy a distinctive and rapidly expanding region of chemical space, enabling a wide range of applications in therapeutics, nutrition, cosmetics, and materials science.

## Graphical abstract

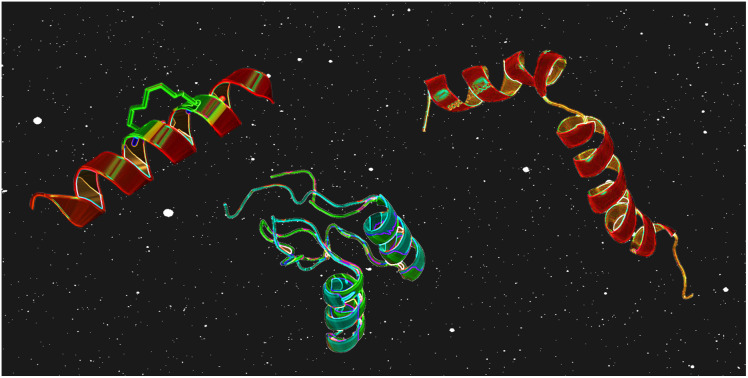

## 1 Introduction

Chemical space has various definitions that can be classified into two general perspectives that have been recently reviewed and collected.^1^ One set of definitions is focused on the “total number of chemical compounds” that can exist. Other definitions, in addition to the N-number of compounds, include a set of M-descriptors that generate a multi-dimensional space in which the compounds are located, like the concept of chemical space proposed by Virshup *et al.*^2^ The latter view of the chemical space gives rise to the high-dependence of chemical space on the number and type of descriptors that are used to construct or define the space. Such variability of dependence has led to the concept of “chemical multiverse” which has been defined as the group or collection of chemical spaces of a given set of compounds, each space defined by a specific set of descriptors.^1^

Since, in practical applications, the chemical space depends on the N-number and type of chemical compounds that are being studied, it would be appropriate to refer to “chemical subspaces”, for example, the subspace of drug-like organic compounds, metal-containing drugs, food chemicals, materials, and peptides.

Chemical space analysis, in particular, visual and qualitative/quantitative analysis, has multiple applications such as library design, compound selection, structure–property relationships, and chemical diversity analysis. To date, most common applications of chemical space are focused on small molecules (organic compounds) and peptides, with emerging and growing applications in other areas (such as food chemicals, materials, and organometallic molecules).^3^

Peptides as bioactive compounds are attractive in research areas like drug discovery, owing to their unique properties. Their high specificity and affinity to interact with targets make them ideal for certain applications in multiple fields, including therapeutics, material science, cosmetics, food chemistry, and biotechnology.^4–9^ The first proof of peptides' potential is given by the medical use of insulin in 1922 for type 1 diabetes, starting their increasing relevance in modern biomedicine.^6^ The architectural diversity and operationally versatile characteristics of peptides further contribute to their constantly increasing importance in these areas.^10,11^

These extensive advantageous characteristics have led the researcher to explore the combinatorial potential of peptides, as their sequence variability generates a virtually limitless number of possible structures.^12^ The peptide's features stem from the amino acid chain's length and conformation, which can be composed of canonical amino acids (cAAs) or can be further expanded by incorporating non-canonical amino acids (ncAAs), synthetic modifications, and post-translational modifications (PTMs).^13^ Various elements serve to create a vast and versatile chemical domain that is under continuous exploration and expansion.^14^

Considering that a peptide comprises a series of amino acid monomers linked in a linear sequence, the range of potential peptides increases dramatically as the length extends.^10,11^ This theoretical diversity establishes the basis of peptide chemical space for discovering new bioactive molecules and refining potential therapeutic options.^12^ The extensive nature of this space encounters several difficulties regarding synthesis, stability, and the feasibility of practical application. By mapping peptide chemical space, it is possible to seize its potential for developing new drugs, biomaterials, and functional molecules tailored to specific applications.

In the past few years, the increased interest in studying peptides from various applications (including the deep knowledge and characterization of bioactive peptides that are toxic compounds – biotoxins) has attracted the attention of the community to chart the chemical space of peptides.^12,15,16^ Similar to the exploration of the chemical space of small organic compounds, a key aspect in the charting of the chemical space of peptides is the nature/type and number of descriptors used to define the space, along with the number and type of specific peptides under study.

The goal of this review is to survey the progress on the exploration and expansion of peptide chemical space. The review is organized into five main sections. After this Introduction, we discuss practical applications of peptides. Section 3 presents an analysis of the size of the chemical space of peptides. The next section discusses approaches to systematically explore – quantitatively and visually – and expand the chemical space of peptides. The last section presents concluding remarks. The review focuses on short and long peptides (with < 50 amino acids).

## 2 Practical applications of peptides

Due to their structural versatility, biocompatibility, and ease of synthesis, small peptides have gained increasing interest across various fields ranging from biomedical sciences to food technology and materials engineering. Hereunder, we discuss exemplary practical applications highlighting their therapeutic and industrial potential.

### 2.1 Drug discovery: novel peptide therapeutics

In drug discovery, small peptides serve as promising candidates for novel therapeutics due to their high specificity and low toxicity.^17,18^ Since the discovery of insulin in 1922, peptide drugs have been developed to treat a wide range of diseases, including cancer, immunological diseases, metabolic disorders, viral infections, cardiovascular diseases, and other chronic diseases.^19,20^ The advancement in peptide technology over the past decades is changing the drug discovery landscape.

Insulin was isolated by Banting and Best from dog pancreas and later from bovine sources, after which it was further purified and its amino acid sequence determined. In 1982, human recombinant insulin was produced for the first time in *E. coli* and yeast. Today, rapid-acting insulin analogs are being developed to optimize glycemic control.^21^

Pharmacologically active peptides are hard to formulate as drug products, as compared to small molecules, due to the various challenges in administration and delivery of therapeutic peptides into cancer cells and tumor sites. Typically, peptide drugs (with cAAs) exhibit shorter circulation half-lives, lower cell permeability, and typically higher rates of enzymatic degradation. Nevertheless, therapeutic peptides with cAAs and ncAAs have the advantage of high target specificity and low toxicity. Overcoming their current limitations will lead to safer and more effective drugs.^19,22^

**Fig. 1:**
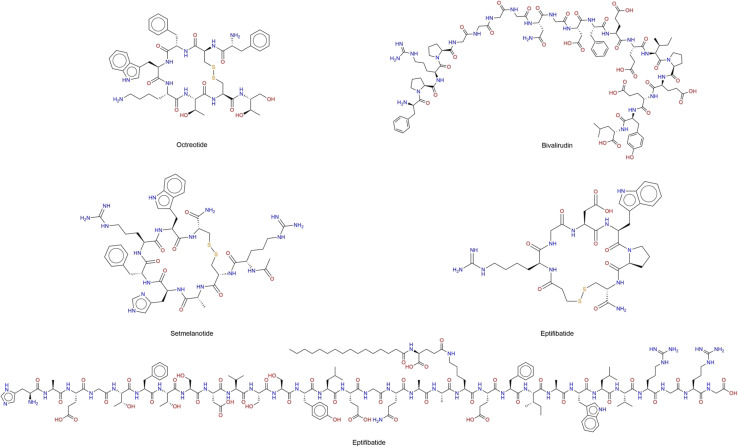
Chemical structure of selected peptides approved by the Food and Drug Administration (FDA) of the United States for clinical use. Linear amino acid sequences (one-letter code) of selected therapeutic peptides: octreotide (FCFWKTCT), bivalirudin (FPRPGGGGNGDFEEIPEEYL), setmelanotide (RCAHFRWC), etifibatide (CXGDWPC), and liraglutide (HAEGTFTSDVSSYLEGQAAK(^1^)EFIAWLVRGRG), where K(^1^) denotes a lysine residue acylated with palmitoyl-γ-Glu (palmitic acid attached *via* a glutamic acid linker).

The development of therapeutic peptides has followed diverse paths, illustrating the main challenges and solutions in the field. An emblematic case is that of the incretin peptide GLP-1, initially limited by its rapid degradation in blood, which led to the design of analogs resistant to the DPP-4 enzyme and with structural modifications that prolonged their half-life, giving rise to successful drugs such as liraglutide and semaglutide (Fig. 1). Glucagon was first isolated in 1923 and approved by the FDA in 1960 for treating severe hypoglycemia. In 1982, the glucagon gene was identified in the Atlantic anglerfish, enabling the discovery of mammalian glucagon genes and the production of recombinant glucagon in bacteria. By the late 1990s, recombinant glucagon became commercially available, yet it retained the same stability issues.^23,24^

The use of partially or fully substituted l-amino acids with d-amino acids is a strategy to decrease proteolytic cleavage and lower immunogenicity. An example is octreotide, an FDA-approved octapeptide (Fig. 1), that is an unnatural d-enantiomer modification, which is used in the treatment of gastrointestinal tumors. The development of octreotide traces back to the discovery of somatostatin and the desire to harness its inhibitory effects on hormone secretion, while overcoming somatostatin's extremely short half-life. Researchers adopted a strategy of peptide analog design, selecting shorter cyclic peptides that could maintain receptor binding yet resist proteolytic degradation.^25^ In particular, they introduced d-amino acids and chemically constrained the peptide by cyclization (disulfide bridge) to increase metabolic stability and receptor affinity. Lead optimization employed structure–activity relationship (SAR) studies to refine receptor subtype selectivity and pharmacokinetic properties. The resulting octreotide with an enhanced half-life, high affinity for somatostatin receptor subtypes 2 and 5, and improved bioavailability.^18,20,26^

Significant achievements have been made in the efficacy and selectivity of therapeutic peptide delivery. The bioavailability and stability of therapeutic peptides have been increased due to the development of several formulation and delivery methods, including prodrug approaches, direct chemical modifications, applying special drug delivery systems, co-administration of enzyme inhibitors and absorption enhancers, because free peptides are not systematically stable without modifications.^27,28^ For example, peptide cyclization is a structural manipulation where the constrained geometries result in dramatically reduced proteolytic degradation by amino and carboxypeptidases. Octreotide is the stable analogue of the parent peptide, somatostatin. Similarly, rational structural optimization played a central role in the design of bivalirudin (Fig. 1), which was developed through a structure-based approach aimed at creating a safer and more controllable anticoagulant than heparin. Inspired by the natural thrombin inhibitor hirudin, researchers used the crystal structure of the thrombin–hirudin complex to engineer shorter synthetic analogs. Peptide synthesis and biochemical assays led to the identification of bivalirudin, a 20-amino-acid peptide that binds reversibly to thrombin's active site and exosite I, providing potent yet transient anticoagulation.^29,30^ While eptifibatide (Fig. 1) was engineered from the snake venom peptide barbourin, using SAR-guided optimization and cyclic peptide synthesis to enhance receptor selectivity, stability, and pharmacokinetic properties.^31^

Setmelanotide (Fig. 1) and zilucoplan (chemical structure not shown) exemplify the application of rational peptide design and optimization in modern drug discovery. Setmelanotide, an eight-amino acid agonist of the melanocortin-4 receptor (MC4R), was developed through SAR studies and receptor binding assays to enhance potency, selectivity, and signaling bias toward Gs-mediated pathways involved in appetite regulation. Similarly, zilucoplan, a synthetic 15-residue macrocyclic peptide, emerged from an mRNA display screening platform that identified high-affinity binders to complement component C5. The lead sequence was optimized through solid-phase peptide synthesis, structural cyclization, and lipophilic modification to improve stability and pharmacokinetic properties, demonstrating how diverse engineering strategies contribute to the development of potent and selective therapeutic peptides.^32–34^

Antibiotics invented or discovered like penicillin by Alexander Fleming, have been used as a wonder drug for almost a century. However, those antibiotics are becoming failures due to their extensive overuse in recent decades, resulting in antimicrobial resistance. This prompted scientists to emphasize other alternatives like ocellatin. Ocellatin is a peptide derived from the skin secretions of *Leptodactylus* genus frogs and has a broad spectrum of antibacterial activities, specifically in Gram-negative bacteria.^35^

Notable progress in the development of vaccines and small molecules with antiviral therapies, the continued emergence and re-emergence of viral outbreaks, along with rising antiviral resistance, have driven researchers to constantly seek new antiviral candidates.^36^ Hence, antiviral peptides that mainly originate from antimicrobial peptides with antiviral activities can be prospective antiviral agents to fight viral infections. Antiviral peptides typically are short (12–50 amino acid residues), and hydrophobicity is likely to be a key characteristic for antiviral peptides to target enveloped viruses. These antiviral peptides act against enveloped viruses by interrupting the fundamental stages of their life cycle of entry, synthesis, or assembly. Naturally, antimicrobial peptides with antiviral properties have been found in almost all multicellular organisms, like plants, animals, mammals, and microbes. It is important to mention that marine organisms are highly regarded reservoirs of pharmacologically active molecules, including peptides. Marine organisms biosynthesize structurally unique and bioactive compounds as an adaptive response to the harsh, highly competitive, and physiologically demanding conditions of the environment; conditions that markedly contrast with those of terrestrial ecosystems. These extreme ecological pressures drive the evolution of potent and functionally diverse molecular architectures.^37,38^

Marine-derived cyclic and linear peptides have significantly advanced our understanding of ion channel modulation, antimicrobial activity, cytotoxic mechanisms, and other pharmacologically relevant properties, thereby positioning marine peptides as promising candidates for innovative therapeutic development.^37^

An example of the antiviral peptide is the HIV-1 targeting human neutrophil peptide HNP-1 exhibits an indirect mechanism of action by binding both the viral envelope glycoprotein Env and host cell surface molecules, including CD4 and co-receptors, in a manner that is independent of glycan interaction and serum components.^36^ Additionally, its capacity for oligomerization or conformational rearrangement may sterically hinder the fusion process.^27,39,40^

### 2.2 Food and nutrition: functional peptides with specific health benefits

In recent years, food has been increasingly recognized not only as a source of essential nutrients, but also as a reservoir of biologically active compounds capable of promoting human health and enhancing physiological functions. Among these compounds, bioactive peptides (BAPSs) (short sequences of 2 to 20 amino acid residues with molecular weights ranging from 0.4 to 2 kDa) have attracted considerable attention due to their diverse health-promoting properties. Although less common, longer peptides such as lunasin (a 43-residue peptide from soy) have also been identified, exhibiting anticancer and hypocholesterolemic effects.^41,42^

BAPS are typically released from parent proteins during enzymatic hydrolysis (*e.g.*, using trypsin, pepsin, alcalase) or through microbial fermentation. In addition to their role in basic nutrition, food-derived protein hydrolysates can exert immunomodulatory, anticancer, antihypertensive, antioxidant, antimicrobial, antidiabetic and anti-inflammatory effects. BAPs have been isolated from a wide range of sources including milk, egg, fish, soybean, rice, pea, oyster, mussel, chlorella and spirulina.^43–45^

Microalgae have emerged as sustainable, protein-rich organisms with the ability to synthesize a wide array of primary and secondary metabolites. Their high content of essential amino acids, combined with a rich profile of bioactive compounds, positions microalgae as promising therapeutic agents and valuable sources of functional food ingredients. For instance, *Arthrospira platensis* (spirulina), a blue–green microalga consumed globally as a nutraceutical supplement, displays notable anti-inflammatory activity through the suppression of pro-inflammatory cytokines and gene expression.^46^ Microalgae-derived peptides have demonstrated a wide range of bioactivities, including antihypertensive, antioxidant, anti-inflammatory, anticancer, antibacterial, antiallergic and antidiabetic effects. These peptides can be incorporated into various functional food products such as beverages, baked goods, pasta, yogurts and sports supplements, offering enhanced nutritional profiles without compromising sensory quality.^47^

A particularly relevant class of bioactive peptides is antimicrobial peptides (AMPs). AMPs display broad-spectrum antimicrobial activity, unique structural features and mechanisms of action that reduce the risk of drug resistance. Beyond disrupting bacterial membranes, some AMPs can penetrate cells and inhibit nucleic acid or protein synthesis, showing potential against multidrug-resistant strains.^48^

Fermented foods, especially in Asian countries, are another important source of bioactive peptides. Traditional fermented products like soybean (*e.g.*, sufu), fish, and milk derivatives are rich in peptides with antioxidant, antihypertensive, antimicrobial, antidiabetic, and anticancer properties.^49^ Fermentations enhance not only shelf life but also flavor, texture, and nutritional value, due to proteolytic activity that releases bioactive peptides during ripening. Despite their benefits, some protein hydrolysates, particularly from soy, may generate bitter-tasting peptides during enzymatic hydrolysis, affecting palatability. Interestingly, the protease produced by *Mucor* species, used in fermented products like sufu, can degrade soybean protein without producing bitter peptides, while still generating bioactive polypeptides.

Fish proteins also serve as a high-quality source of peptides, particularly due to their content of essential amino acids and polyunsaturated fatty acids. Fermented fish products have been reported to exhibit antioxidant and ACE-inhibitory activity, largely due to the presence of low-molecular-weight peptides formed during processing.^48,49^

Peptides obtained from various dietary proteins have been shown to exhibit diverse biological activities, including immunomodulatory, anticancer, antihypertensive, antioxidant, anti-inflammatory, mineral-chelating, lipid-lowering, bone-protective, and antimicrobial properties.^8,50^

Modulating immune function through dietary components has proven to be a practical and effective approach; additionally, the identification of new immune-modulating peptides derived from food proteins may offer added benefits in dietary-based therapies.^50^

### 2.3 Cosmetics and materials: peptides for biomaterials, nanotechnology, and skin penetration enhancers

The field of medical aesthetic skin care includes a vast array of ingredients and topical formulations, emphasizing the importance of a careful and evidence-based selection process. Evaluating the quality of scientific support for manufacturer claims is essential, including *in vivo* and *in vitro* studies that validate ingredient efficacy and practitioner preferences; these factors are key in determining product use, although not exhaustive. Peptides have advantages over small chemical molecules in specificity and selectivity, but they often have poor ability to penetrate skin.^51^ Due to their multifunctional and regenerative capabilities, peptides have become a topic of growing scientific interest. Their biological activity depends largely on their structure and includes antioxidant, anti-aging, moisturizing, promoting collagen production, and wound-healing effects.^52^

**Fig. 2:**
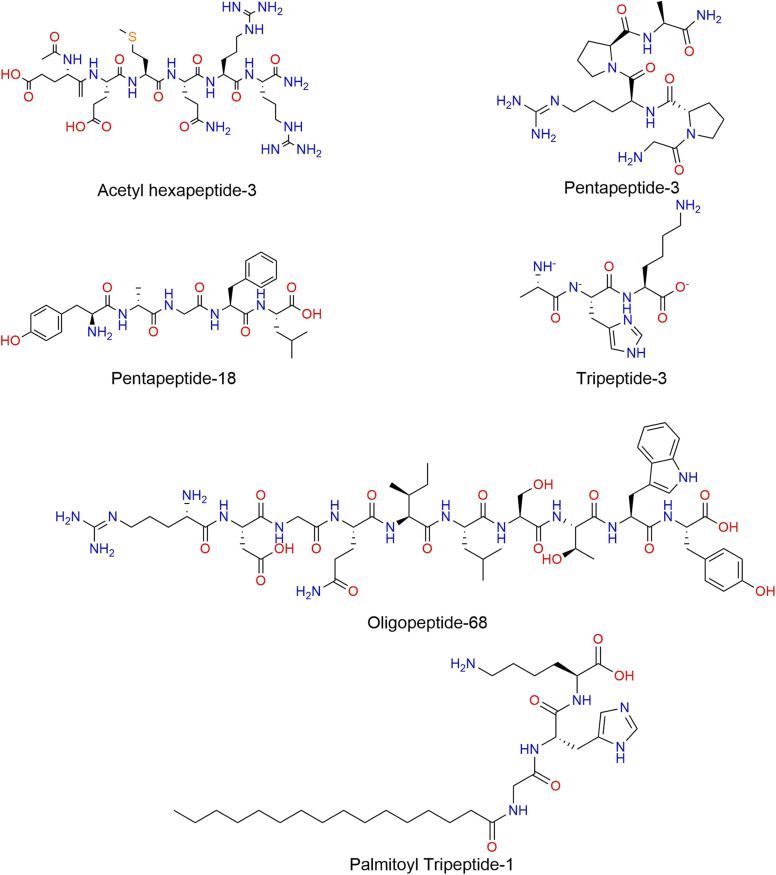
Examples of therapeutic and functional peptides commonly used in cosmetic formulations, discussed in the manuscript.

Peptides can be classified by their mechanism of action into several functional categories: signal, carrier, neurotransmitter-inhibiting, enzyme-inhibiting, and antimicrobial peptides. The signal peptides stimulate the synthesis of collagen and elastin, one of the first cosmetic signal peptides to demonstrate this effect was palmitoyl peptide (Fig. 2).^4,53^

Carrier peptides facilitate the delivery of essential trace elements involved in enzymatic activity and tissue repair, contributing to improve skin elasticity, the first commercialized carrier peptide was formulated to deliver copper, which is a trace element necessary for wound healing.^38^ Neurotransmitter-inhibiting peptides act by reducing neurotransmitter release, a process responsible for muscle contraction. By modulating this mechanism, they help diminish the appearance of fine lines and wrinkles. Acetyl hexapeptide-3, pentapeptide-3, pentapeptide-18, and tripeptide-3 (Fig. 2) exhibit neuro-suppressive abilities and are referred to as neurotransmitter peptides.^14,54^ Enzyme-inhibiting peptides prevent collagen degradation by inhibiting specific enzymes, thereby maintaining the integrity of skin structure. An example that is used in skincare is oligopeptide-68 (Fig. 2). Antimicrobial peptides defend against pathogens, including bacteria, fungi, and viruses, by compromising microbial membrane integrity. Myristoyl tetrapeptide-13 (Fig. 2) is an example of a synthetic lipopeptide with potent antimicrobial activity.^54,55^

The amino acid sequence of a cosmetic peptide plays a crucial role in determining its effects on the skin. Each amino acid in a peptide sequence contributes to the shape and charge of the molecule, therefore determining how the peptide interacts with the receptors and enzymes and how it diffuses through the lipid layer. Considering this, peptides containing amino acids with a positive charge, such as lysine, bind with a higher frequency to the membrane if they are located at the extremities of the sequence. Nevertheless, peptides composed of less hydrophobic, polar residues are much less likely to adsorb to membranes than phenylalanine-based peptides.^53^

Peptides used for cosmetic applications can be combined with zinc sulfate to enhance their antimicrobial effect and lesion-healing. Also, peptides can be formulated with vitamin E in antioxidant formulations, addressing structural and oxidative damage. Peptides contribute to the reduction in oxidative stress in the skin by scavenging free radicals through different pathways, resulting in delaying the skin's aging process.^51,52^

Recent research has shifted toward evaluating not only the biological activity of peptides, but also their bioavailability and formulation stability. While peptides offer multiple advantages as active ingredients in cosmetic applications, the development of new formulations is often constrained by issues related to stability, solubility, and skin permeability. One of the main challenges in the manufacturing process is preserving the structural integrity and bioactivity of peptides, which may be compromised by factors such as interactions with other formulation components, pH changes, temperature variations, and processing methods. To address these challenges, it is crucial to select excipients that are chemically inert or minimally reactive to reduce the risk of degradation.^51,52^

Development of bioactive peptides as safe and effective skin-care products, including dermatological applications such as wound healing, requires an understanding of their interaction with the various components present in the skin. Preclinical formulation of cosmetic and dermatological creams by observing the epidermal properties after application allows for a more complete understanding of the safety and efficacy of the product.^53,55^

## 3 The size of the chemical space of peptides

Currently, peptides offer many opportunities in therapeutic and nontherapeutic areas, such as drug discovery, materials science, cosmetics, nutrition, and synthetic biology.^5,53^ Versatility stems from the colossal combinatorial diversity of amino acids in peptides, highlighting the theoretically limitless peptide sequences conceivable.^54^ The variety of potentially viable peptides has escalated by accounting for ncAAs, synthetic amino acids, and PTMs.

A peptide canonically consists of a linear chain with an “*n*” number of amino acids, where each link in the chain comes from a pool of 20 possible cAAs or proteinogenic amino acids.^56^ The length of a peptide starts with two amino acids and has an arbitrary cut-off, typically set below 50 or 100 residues.^57–59^ The number of possible sequences for a given peptide length is obtained by the formula:^15^*P*(*n*) = *A*^*n*^where *P*(*n*) defines the total peptide sequences, *A* represents the count of selected amino acids as building blocks (like 20 proteinogenic forms), and “*n*” designates the peptide's length.

The count of theoretical chains escalates exponentially as the target protein length augments, and with it the possible peptides for exploration. This group of peptides displays all the peptide combinations up to a certain length limit (usually between 50–100 residues). This chemical space provides a multitude of potential therapeutic peptides, crucial for drug discovery.^8^ However, the number of peptides expands indefinitely when we add the non-canonical, synthetic amino acids and PTMs.^60^ This trend can be appreciated, for instance, in the work of Orsi and Reymond,^11^ who reported a virtual library comprising approximately 1 × 10^60^ peptide-like molecules, which were generated from the assembly of 100 commercially available peptide and peptoid building blocks into linear and cyclic oligomers of up to 30 units. This represents nearly 21 orders of magnitude more than the number of theoretical peptides of equivalent length derived from the 20 cAAs. The diversity increases even further when considering more building blocks like those 545 catalogued in the NORINE database,^61^ which also currently contains 1744 unique entries of nonribosomal peptides. As new peptides and monomers continue to be discovered and incorporated into such databases, the accessible peptide chemical space expands.^59^Table 1 summarizes examples of peptide-related databases that provide a sense of the currenlty explored chemical space of peptides.

**Table 1:** Examples of peptide-related databases and computational resources for peptide research

Database or resource	Description	Number elements	Website	Ref.
PDB (protein data bank)	An open-access repository that archives three-dimensional structural data of biological macromolecules determined mainly by X-ray crystallography, NMR spectroscopy, and cryo-EM.	>100 000 entries	https://www.rcsb.org/	62
PeptideAtlas	Repository of experimental proteomics data (mass spectrometry)	6 636 295 537 peptide spectrum matches, 114 builds	https://peptideatlas.org/	63
UniProt	Comprehensive protein/peptide resource	253 635 358 entries	https://www.uniprot.org/	64
Peptipedia	Integrates >30 peptide databases (antimicrobial, anticancer, *etc.*) in a unified resource	3 983 654 peptides	https://peptipedia.cl/	65
NIST	Peptide mass spectral libraries	>4 300 000 spectra; 1 260 000 entities	https://chemdata.nist.gov/dokuwiki/doku.php?id=peptidew:start	66
IEDB	The immune epitope database	>1 600 000 peptidic epitopes	https://www.iedb.org/	67
ProteomicsDB	Human protein expression and PTM data	684 691 human peptides	https://www.proteomicsdb.org/	68
PRIDE	The PRoteomics IDEntifications database	>500 000 peptides (PRIDE crosslinking)	https://www.ebi.ac.uk/pride/	69
SATPdb	Structurally annotated therapeutic peptide database	37 100 entries	https://webs.iiitd.edu.in/raghava/vaxinpad/index1.html	70
DRAMP 4.0	Curated data repository of antimicrobial peptides	30 260 entries	http://dramp.cpu-bioinfor.org/	71
DBAASP v3	Antimicrobial peptides: Activity and structure	>23 000 monomer, multimer 420, multi peptide 236	https://dbaasp.org/home	72
CAMP_R4_	Collection of antimicrobial peptides	>24 000 sequences	https://camp.bicnirrh.res.in/	73
Propedia	Database of peptide–protein interactions	>19 000 peptide–protein complexes	https://bioinfo.dcc.ufmg.br/propedia/	74
CancerPDF	Cancer peptidome database of bioFluids	14 367 experimentally validated peptides	https://webs.iiitd.edu.in/raghava/cancerpdf/	75
PepBDB	Peptide binding database	13 299 structures of peptide-mediated protein interactions	http://huanglab.phys.hust.edu.cn/pepbdb/	76
HORDB	Hormone peptide database	7390 peptide hormones. Includes structural, functional, and bioactivity data	http://hordb.cpu-bioinfor.org/	77
CancerPPD2	A repository of experimentally verified anticancer peptides and anticancer proteins	6521 entries	https://webs.iiitd.edu.in/raghava/cancerppd2/	78
CAD v1.0	Cancer antigenic peptide database	6000 simulated neopeptides; 800 cancer antigens	http://cad.bio-it.cn/	79
APD6 (antimicrobial peptide database)	Comprehensive antimicrobial peptide database (original version)	5680 peptides	https://aps.unmc.edu/	80
NeuroPep 2.0	Neuropeptides	11 417 unique neuropeptide entries	http://isyslab.info/NeuroPep/	81
DADP	Database of anuran defense peptides	2571 entries	http://split4.pmfst.hr/dadp/	82
PepLife	Half-life information for therapeutic peptides (experimental data)	2172 entries	https://webs.iiitd.edu.in/raghava/peplife/	83
Norine	Database of non-ribosomal peptides	1744 peptides	https://norine.univ-lille.fr/norine/	84
MBPDB	Bioactive peptides derived from milk proteins	691 bioactive peptides sequences	https://mbpdb.nws.oregonstate.edu/	85
THPdb	Therapeutic peptide database	852 entries	https://webs.iiitd.edu.in/raghava/thpdb2/	86
BIOPEP-UWM	Database of bioactive peptides	7857 entries	https://biochemia.uwm.edu.pl/en/biopep-uwm-2/	87

### 3.1 Comparison of theoretical *vs.* practically synthesizable peptides

While the theoretical total number of peptides (peptide chemical space) is astronomically large, we can distinguish between unexplored and explored chemical spaces, each containing bioactive and non-bioactive compounds.^59,88^ Comparing the vast unexplored chemical space with explored or known space, represented by accessible peptides in libraries;^8^ uncovers that the most promising active peptides are still undiscovered, and eligible for synthesis and experimentation.^89^ Instead, the variety of peptides that can be feasibly created is constrained by numerous variables, which fluctuate based on the method of production and the attributes of the amino acid residues. These limitations are present in synthesis methods, folding stability, cost, and efficiency.

Chemical and enzymatic approaches to synthesizing longer peptides face limitations related to peptide length, purity, and complexity. As peptide length increases, yields tend to decrease, and synthesizing complex sequences often requires advanced techniques. Additionally, synthesizing peptides with PTMs is challenging, for instance, in addressing aggregation issues. These difficulties can result in low yields, reduced purity, and, in some cases, failure to obtain the desired peptide.^90^

Some peptide sequences tend to aggregate or misfold, making them challenging to synthesize, isolate, and study. The difficulties associated with synthesizing certain peptides are encapsulated in the term “difficult peptides”, introduced in the 1980s. These peptides share a common characteristic which is a high propensity for aggregation. This phenomenon arises from significant inter- or intra-molecular β-sheet interactions, which promote aggregation during synthesis. These structural interactions are stabilized by hydrogen bonds along the peptide backbone, making certain sequences prone to aggregation.^91^

The synthesis of longer and more complex peptides gets costly and time-consuming, thus restricting the feasible peptide chemical space. Improvements in peptide synthesis methods improve their scalability and yield. Advancements in reactive materials like resins, amino acid derivatives, and coupled reagents; along with better purification methods, have been vital for cost-cutting, boosting yield and purity, and facilitating complex modifications. As a result, peptide companies offer peptides of 15 to 20 amino acids, including those with natural and non-natural or modified residues, at relatively low costs.^92^ Thus, while the theoretical number of peptides is nearly limitless, practical synthesis and study are constrained by technical and economic factors.

Moreover, enzymatic synthesis, while potentially more efficient, but costly cofactors limit large-scale production. These reagents limit the variety of studyable peptides, showing a significant gap between the theoretical peptide chemical space and the subset accessible for experimental exploration.^93^

Additionally, availability of building blocks, especially non-proteinogenic amino acids (npAAs), limit the space for practically made peptides. Not all npAAs have been made yet,^94^ and their chemical synthesis have their own challenges due to several issues such as stereoselectivity and low production yields. Many amino acids are chiral, with 19 of the 20 canonical ones containing at least one chiral center, excepting glycine, which is the only achiral cAAs. Among them, isoleucine and threonine each have two chiral centers at α- and β-carbons.^95^ Stereochemistry is key to defining the structural and functional properties of peptides.^96^ Variations in the spatial arrangement of atoms influence backbone conformation, side-chain orientation, and overall molecular dynamics, which impact biological recognition, binding affinity, and functional selectivity.^96–98^ From a broader perspective, stereochemical diversity represents an additional dimension of the peptide chemical space (stereochemical space),^99^ enabling the exploration of novel structural motifs and conformational states beyond those encoded by natural amino acids. Expanding this stereochemical landscape not only enhances the potential for discovering peptides with improved stability, bioavailability, or activity but also provides a richer framework for *in silico* modeling and rational design of bioactive peptide scaffolds.^100,101^

Beyond stereochemical variation, molecular diversity also arises from the incorporation of non-canonical and chemically modified amino acids. While stereochemical changes influence the conformational landscapes of amino acids, the introduction of new monomers expands peptide chemical space by adding novel functional groups, backbone structures, and reactivities. Together, these complementary strategies—stereochemical diversification and amino acid development—offer enhanced opportunities for designing peptide structure, dynamics, and function.

### 3.2 Incorporation of non-canonical and synthetic amino acids, post-translational modifications

The chemical space of peptides has greatly expanded through the incorporation of ncAAs and PTMs as building blocks.^92,102^ ncAAs, also known as unnatural or npAAs, non-standard amino acids (nsAAs), or unnatural amino acids (unAAs),^14^ come from different types of amino acids not present genetic code.^103^ Most ncAAs are synthesized chemically or semi-synthetically, while only a few can be produced through natural *in vivo* pathways.^103^ Their inclusion dramatically expands the chemical space of peptides, offering unprecedented opportunities to fine-tune peptide structure-related functions. For example, amino acids such as selenocysteine and pyrrolysine enhance peptide reactivity and structural versatility.^92,104,105^ Moreover, modified amino acids can improve peptide pharmacokinetic properties and increase thermal stability or resistance to enzymatic degradation.^106^

PTMs, like phosphorylation, acetylation, methylation, and glycosylation, contribute to the expansion of peptidic chemical space.^107^ Those modifications extend their potential application by expanding their functions, stability, and interactions.^108–112^ Thus, the introduction of PTMs is relevant for therapeutic purposes, mimicking natural alterations by their chemical or enzymatic addition.^110,113^ On the one hand, chemical synthesis provides a uniform introduction of particular modifications at specified sites within a protein or peptide of interest. Advancements in peptide ligation methods and efficient coupling agents now enable the synthesis of long peptides with tailored modification and PTMs, therefore influencing their structural integrity, functional properties, and overall stability.^114–117^

On the other hand, the *in vitro* peptide enzymatic modifications often lead to heterogeneous products due to limited specificity and insufficient control over the extent of modification. Besides, complementary biological strategies, such as genetic code expansion technology, allow precise incorporation unAAs into target proteins by utilizing engineered orthogonal aminoacyl-tRNA synthetase/tRNA pairs.^116–119^ This technique permits including diverse site-specific PTMs into recombinant proteins, including modifications such as acetylation, methylation, phosphorylation, and nitration. This method relies on the accessibility of an orthogonal tRNA synthetase specifically designed for the intended modification. However, the efficiency of this approach decreases when incorporating multiple PTMs within a single peptide or protein, presenting significant challenges for achieving large-scale combinatorial modifications.^116–119^

Head-to-tail macrocyclization is a naturally present PTM that stabilizes the protein and peptide fold, enhancing thermal stability and resistance to exoprotease proteolytic degradation.^120^ In nature, cyclic peptides are present in bacteria, fungi, plants, and marine species, displaying remarkable diversity in shape, size, and chemical composition.^120,121^ Their therapeutic potential arises from their capacity to inhibit enzymes, interfere with protein–protein interaction, modulate cell signaling, and regulate immune responses.^121^ Their exceptional stability and selectivity make them ideal candidates for drug design. Additionally, cyclic peptides serve as crucial tools for drug discovery, functioning as molecular probes for detecting protein function, disease mechanisms, or therapeutic targets. Novel developments in methodologies including solid-phase peptide synthesis (SPPS), chemoenzymatic synthesis, and orthogonal protection strategies, have enhanced the specificity and complexity in the fabrication of cyclic peptides.^121^

Integration of ncAAs, cyclic peptides, and PTMs notably widens the peptide chemical multiverse, facilitating the creation of functional peptides beyond the capabilities of standard amino acids.

## 4 Exploration of the known chemical space of peptides

The exploration and systematic representation of peptide chemical space have become essential in both bioinformatics and chemoinformatics. Peptides occupy a unique position between small molecules and proteins, exhibiting complexity in their sequences, conformations, and physicochemical properties, which makes them highly diverse. To enable the rational exploitation of this diversity, a variety of computational frameworks, databases, and molecular representations have been developed. These resources offer standardized notations, molecular fingerprints, and structural encodings, facilitating the study and comparison of peptide structures, analog identification, property prediction, and machine learning-based modeling. Table 2 summarizes representative tools and resources, highlighting their main functions and the types of chemical or structural information they provide. The subsequent sections further discuss the principles and applications of selected methods in the context of peptide informatics and chemical space exploration.

**Table 2:** Selected bioinformatic and chemoinformatic resources for exploring peptide chemical space^a^

Tool name	Website (when available)/short description	Ref.
CHUCKLES (chirality-oriented chemical representation)	Method that interconverts peptide or peptoid sequences with SMILES, enabling both sequence- and structure-based searches, including branching and cyclic structures	122
SCSR (self-contained sequence representation)	Method that encodes amino acid sequences with side-chain chemical info for modeling	123
MAP4 (MinHashed atom-pair fingerprint up to a diameter of four bonds)	A molecular fingerprint that combines atom-pair concepts with MinHashing to efficiently represent both small molecules and large biomolecules. It captures structural and topological features up to four bonds apart, enabling fast and scalable molecular similarity searches across diverse chemical spaces. https://github.com/reymond-group/map4	124
MAP (modification and annotation in proteins)	Format extends the traditional FASTA format by enabling annotation of modified residues, post-translational modifications, binding sites, mutations, and protein metadata. https://webs.iiitd.edu.in/raghava/maprepo/	125
ECFP (extended connectivity fingerprint)	A circular molecular fingerprint that encodes atomic neighborhoods based on connectivity patterns. Widely used in cheminformatics for similarity searching, clustering, and QSAR modeling, ECFP captures local structural features around each atom to represent molecular topology in a compact, numerical form. https://docs.chemaxon.com/display/docs/fingerprints_extended-connectivity-fingerprint-ecfp.md	126
HELM (hierarchical editing language for macromolecules)	Enables standardized representation of complex biomolecules, including proteins, nucleotides, and antibody-drug conjugates	127
PLN (protein line notation)	A linear, text-based representation for describing protein and peptide sequences in a compact, machine-readable format. PLN encodes sequence information, modifications, and structural annotations, facilitating data exchange, database indexing, and computational analysis of proteins and peptides. http://www.biochemfusion.com/doc/PLN_Guide/PLN_Guide.html	128
3D-VAIF (three-dimensional vector of atomic interaction field)	Structural descriptor method encoding electrostatic and steric atomic interactions for 3D peptide representation	129
MXFP (macromolecule extended atom-pair fingerprint)	A 217-dimensional atom-pair fingerprint designed to encode large molecules (*e.g.*, peptides, macrocycles, natural-products) by representing pharmacophore-group atom-pairs and their topological distances; useful for similarity searching and chemical-space mapping of non-Lipinski or biomolecular compounds. https://github.com/reymond-group/mxfp_python	130
KNIME (the konstanz information miner)	An open-source, modular platform for end-to-end data analytics that enables users to visually build, execute and monitor data workflows—covering extraction, transformation, modelling and visualization—without needing extensive coding. https://www.knime.com/	131
Datawarrior	Software for data analysis and visualization. https://openmolecules.org/datawarrior/	132 and 133
PepINVENT	Peptide design tool extending the REINVENT platform https://github.com/MolecularAI/PepINVENT/	134
PepSMI	A web-tool provided by NovoPro bioscience Inc. that converts a peptide amino-acid sequence (using one-letter codes) into a SMILES (simplified molecular Input line entry system) string, enabling computational representation of the peptide's molecular structure. https://www.novoprolabs.com/tools/prot-sol	135
SignalP	Signal peptide prediction tool. https://services.healthtech.dtu.dk/services/SignalP-6.0/	136
Unipept	Peptide-based metaproteomics tool (biodiversity, biomarker discovery). https://unipept.ugent.be/	137

a Websites are provided when available; otherwise, a short description is included, along with the reference.

### 4.1 Molecular representation

One of the most important considerations for generating representative chemical spaces that serve the purpose of the project's goals (*e.g.*, meaningful chemical spaces) is the appropriate use of the molecular representation and the descriptors that will be the basis to define the (multi) dimensional space. Towards this end, novel representations have been developed to condense structural information of complex molecules, like peptides.^122–130^ For example, hashed fingerprints (*e.g.*, extended connectivity fingerprints (ECFP) and MinHashed atom-pair fingerprint (MAP)) have been distinguished from other unidimensional representations because they can codify the atom connectivity and neighborhoods of complex molecules.^124,126^ However, these kinds of representations could be redundant for polymeric compounds, like large peptides, antibodies, or other kinds of proteins. For polymeric compounds, there have been developed sequence-based representations that take advantage of the molecular redundancy of each compound to simplify their representations. For example, the CHUCKLES notation compacts the structural data of each amino acid into a unique letter, which can codify simple post-structural modifications like cysteine bridges.^122^ Other notations, such as the Hierarchical Editing Language for macromolecules (HELM) and the Self-Contained Sequence Representation (SCSR), can represent more complex amino acid-based compounds like large canonical and non-canonical peptides and antibodies.^123,127^ Interestingly, for polypeptides or proteins with more than 50 amino acids, the most common representations are the PDB entries that contain the coordinates of each atom of the polypeptide/protein and other non-covalent bound atoms (*i.e.*, solvent and ligands atoms), considering the presence of multiple chains, subunits, or multi-domain complexes. However, there exist unidimensional representations to codify protein connectivity information like Protein Line Notation (PLN) and Boehringer Ingelheim Line Notation (BILN) which can convert sequence representation to atom connectivity data without considering three-dimensionality features.^128,138^

**Fig. 3:**
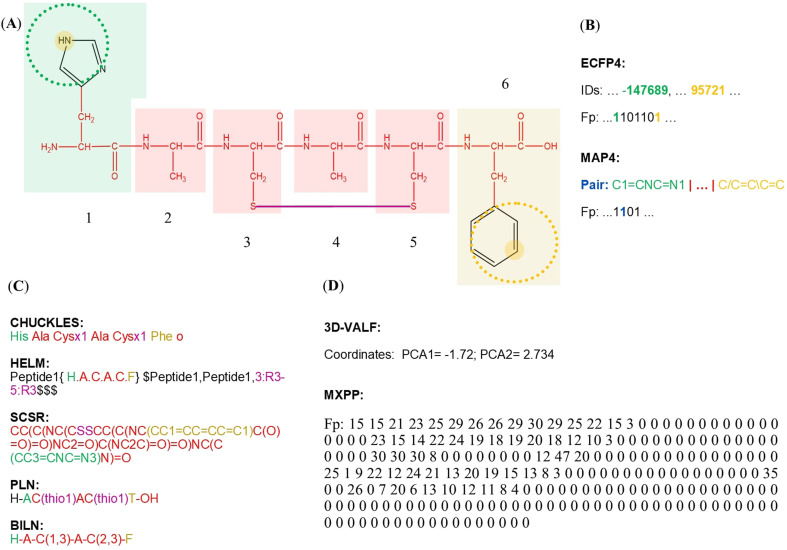
Molecular representations commonly used for peptides. (A) Peptide example. Their chemical structure contains amino acids in a specific order (alanine, cysteine, alanine, cysteine, and threonine), in which their cysteines form a disulfide bond; (B) examples of hashed fingerprints. ECFP4 represents data of each atom in the structure and their connectivity with 2 atoms of distance from these, which is condensed bits after collision methods to avoid redundant connectivity information. A similar example is the MAP4 fingerprint, which uses this same strategy but captures the information on the connectivity of paired atoms; (C) amino acid-based notations. HELM and PLM use the conventional one-letter notation to represent implicitly the amino acid connectivity and use specific codifications to represent out-peptide bonds, like disulfide bonds. However, examples like SCSR, PLN, and BILN representations offer an alternative to represent explicitly the atom connectivity of peptides; and (D) three dimensional-based representations. For example, 3D-VALF uses conformational data to create new vectors from dimensional reduction methods capable of condensing the atom connectivity and property data of whole peptides; MXFP uses fingerprint-based representation to codify the presence of pharmacophoric features. The peptide representations shown in B and D panels are illustrative of the type of data generated by each representation.

Similarly, chemo-bioinformatics representations are innovative strategies to codify the atom connectivity of peptides at the same time as other important features, which has accelerated the development of novel peptide-based molecules with specific features. For example, the three-dimensional vector of atomic interaction field (3D-VAIF) approach captures the information of electrostatic and steric interaction between different types of atoms in peptides.^129^ The macromolecule extended atom-pair fingerprint (MXFP) can describe molecular shapes and pharmacophores.^17,130^ However, one of the major limitations of these representations is their low interpretability for the user, as they consist of alphanumeric codes that can only be understood through mathematical and computational processes (Fig. 3D).

In addition, hybrid fingerprints inspired by different kinds of data, like amino acid sequence, atom-connectivity, and physicochemical properties, have been developed to codify most strictly the chemo-biological features of peptides.^139,140^ Consequently, after the development of more informative molecular representations for peptides, the thin line between traditional bioinformatics and cheminformatics approaches to represent peptides is becoming increasingly blurred. However, there is an unwritten rule of thumb about the use of molecular fingerprints to codify the chemical structure of small peptides (<50 residues) and atom coordinates to represent large peptidic structures (>50 residues).^12^ A few representative representations are illustrated in Fig. 3 for a representative (given) peptide.

### 4.2 Visual representation of the chemical space of peptides

Based on the advances in molecular representations of peptides and the development of novel bio-chemoinformatics descriptors, recent visual representations have enabled illustrating efficiently and intuitively peptidic structure–function relationships and activity-based clusters. This has allowed the creation of intuitive representations to make smart decisions about prospective evaluations for peptide-based compounds.^141,142^ For example, advances in the visualization of atom connectivity similarity–activity relationships have opened new horizons for the optimization process of non-canonical peptides.^143^

### 4.3 Mapping bioactive peptides in chemical space

**Fig. 4:**
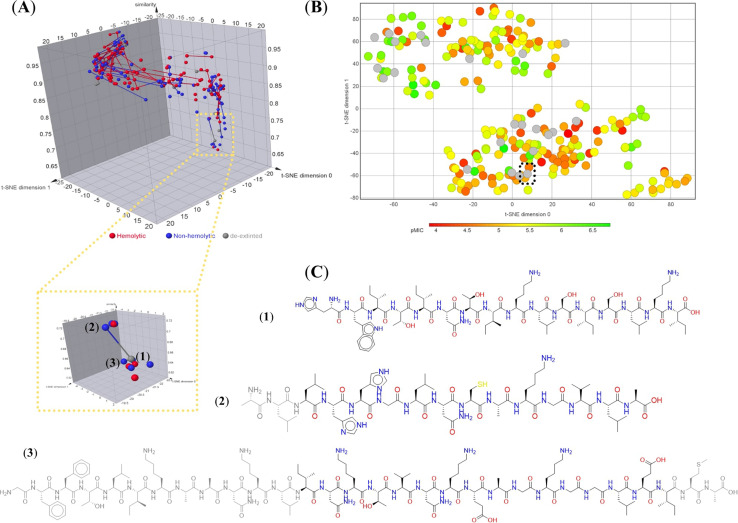
Chemical space representations to study ancient peptides. (A) Structure–activity relationships based on scaffold similarity of representative peptides with hemolytic and non-hemolytic activity. Chemical space based on coordinates and the structure of molecular scaffolds was constructed using a network-based protocol implemented in KNIME software and visualized using Datawarrior software, version 06.04.01.^132,133,146^ Each compound is represented by dots, and their distinctive biological property is represented by different colors. Finally, each compound was linked with other molecules that share a scaffold relationship; (B) visual representation of the chemical space of 223 anti-MRSA non-canonical and canonical peptides. The visual representation was constructed by dimensional reduction (*t*-SNE coordinates) of the ECPF4 fingerprint. Each data point in the graph represents a peptide, and the color in a continuous scale represents the activity values in pMIC from low (red) to high (green). Data points in grey represent peptides with unknown pMIC value (ancient peptides). The dotted line illustrates the chemical space localization of 1 (panel A); (C) representative examples of peptides are illustrated in panel A of this figure (1, HWITINTIKLSISLKI; 2, ALLHHGLNCAKGVLA; 3, GFFTLIKAANKLINKTVNKEAGKGGLEIMA). The atoms colored in grey in each peptidic structure represent the not-aligned moiety with the peptide 1.^147,148^

An illustrative example of the chemical space mapping to decode complex biological properties is shown in Fig. 4. This landscape offers the possibility to study systematically underexplored peptides, like de-extinted peptides, *i.e.*, peptides from the “extinctome” (the proteomes of extinct organisms), which recently has covered particular relevance to developing novel antimicrobial agents.^144,145^Fig. 4 remarks on the flexibility of the chemical space techniques (*e.g.*, using network-based approaches) to identify rapid structure–property relationships in peptides. For example, Fig. 4A illustrates scaffold relationships between each pair of ancient and hemolytic (or non-hemolytic) peptides, and Fig. 4B illustrates chemical space localizations ancient and anti-MRSA (methicillin-resistant *Staphylococcus aureus*) peptides. Thus, remarks about how it is possible to establish structure–hemolytic relationships in peptides and quickly identify the potential anti-MRSA activity of underexplored peptides. Here, it is possible to identify that peptide 1 (Fig. 4C) could have anti-MRSA activity without hemolytic side effects.

### 4.4 Development of machine learning models

Recent advances in machine and deep learning technologies have led to the creation of network-based chemical space representations based on peptidic sequences, which opens up new perspectives on the smart identification of “privileged” scaffolds and representative conserved moieties against specific chemical and biological endpoints. For example, solubility, type of tridimensional folding, cell permeability, bioactivity, toxicity, and hemolysis.^149–151^

The integration of high-throughput screening (HTS) technologies has transformed the early stages of peptide discovery. By enabling the rapid and parallel evaluation of thousands of compounds, HTS platforms generate extensive multidimensional datasets that can be mined to extract patterns linking peptide features with biological responses. These large-scale datasets constitute a critical substrate for the training and validation of AI-based models capable of predicting pharmacologically relevant properties.^152^ Additionally, the implementation of robotic laboratory systems has further advanced this paradigm by ensuring the precision, scalability, and reproducibility of experimental workflows.^152^ Automated liquid-handling robots and integrated analytical instruments can execute complex assay cascades with minimal human supervision, thereby reducing experimental bias and facilitating the generation of standardized, high-quality data amenable to algorithmic modeling.^153^ Complementary to experimental acceleration, virtual screening protocols have become an indispensable component of contemporary peptide discovery pipelines. Virtual screening based on molecular docking, pharmacophore modeling, and quantitative structure–activity relationship (QSAR) analyses enable the rapid triage of chemical libraries, prioritizing peptides with predicted high affinity against specific receptors, favorable physicochemical properties, and adequate ADMET or sensory profiles.^154,155^ Finally, molecular dynamics and quantum chemistry simulations provide a complementary, physics-based dimension to data-driven modeling, which allow the integration of conformational free-energy landscapes, binding stability, and interaction fingerprints—into deep learning frameworks, enhances the capacity to predict structure–function relationships at atomic resolution level.^156^ These hybrid approaches bridge mechanistic simulation and statistical inference, enabling a more comprehensive understanding of molecular determinants underlying pharmacological efficacy and selectivity, as well as the decoding of properties with application beyond pharmacological disciplines.^157–159^

The emergence of AI-powered pharmaceutical laboratories is a paradigm shift toward closed-loop, autonomous discovery ecosystems. These laboratories combine robotic automation with AI to establish self-optimizing experimental frameworks in which predictive models continuously learn from experimental feedback. Such adaptive systems can autonomously design, execute, and analyze experiments, thereby expediting the identification of lead peptides and optimizing their chemical space exploration.^160^

### 4.5 Expansion

Exploring peptide chemical space has emerged as a strategy to identify novel bioactive compounds with enhanced stability, specificity, and pharmacokinetic profiles. Recent advancements have focused on expanding this chemical space through various methodologies, including the design of synthetic combinatorial libraries, incorporation of non-canonical amino acids, the generation of “small-peptidic chemical chimeras”, and generative (*de novo*) computational approaches.^161^ These efforts aim to traverse previously underexplored regions of peptide chemical space, facilitating the discovery of compounds with unique biological activities, higher selectivity, and improved drug-like properties.^162–164^

### 4.6 Peptide enumeration and *de novo* generation

Peptide enumeration and *de novo* generation have generated important contributions in modern peptide science, particularly with the advent of generative models capable of producing diverse and biologically relevant molecules. These approaches leverage advanced machine learning architectures, such as deep generative models and language-based neural networks, to systematically explore and expand the peptide chemical space beyond known structures and/or sequences. For instance, deep generative models can be trained to create novel candidates with desired biological or physicochemical properties, offering a powerful alternative to traditional combinatorial enumeration methods.^165^ Other protocols now incorporate multi-objective optimization strategies, enabling simultaneous design for multiple properties such as structural stability, bioactivity, and membrane permeability, allowing the decodification of sequence-structure–function relationships;^166,167^ Collectively, these methods represent a paradigm shift toward intelligent, data-driven design of peptide therapeutics, enabling the rapid identification of high-potential candidates with tunable properties. To this end, Geylan *et al.* introduced a novel approach, PepINVENT, designed to expand the landscape of peptide therapeutics by incorporating both natural and non-natural amino acids into *de novo* peptide design. PepINVENT enables the generation of peptides with enhanced properties such as binding affinity, plasma stability, and membrane permeability, which are crucial for therapeutic efficacy. This example integrates reinforcement learning algorithms to create and navigate novel regions of the peptide chemical space, incorporating multi-objective optimization strategies inspired by a holistic molecular design approach.^134^

The systematic enumeration of peptide sequences within defined molecular property ranges, such as quantitative estimate of drug-likeness (QED) and toxicity, has become a focal point in computational peptide design. Artificial intelligence algorithms now integrate predictive models that assess key physicochemical properties, enabling the generation of peptide libraries tailored to specific therapeutic profiles, generating the first generation of “focused peptide libraries”.^168,169^ These types of libraries are characterized by their enriched content of biologically relevant and synthetically feasible sequences, typically constrained by user-defined filters such as solubility, stability, net charge, hydrophobicity, and low off-target toxicity. Unlike random or exhaustive combinatorial libraries, focused peptide libraries are constructed to maximize the probability of bioactivity while minimizing redundancy and undesired pharmacokinetic features.^170,171^ In practice, this allows researchers to streamline screening efforts by working with a smaller, high-quality subset of candidates more likely to translate into therapeutic success. Furthermore, the incorporation of domain-specific constraints, such as protease resistance, membrane permeability, organ targeting, and endpoint-specificity into the library generation process allows these datasets to be aligned with specific applications.^172^

On the other hand, maximizing coverage and diversity in peptide chemical space is essential for discovering novel peptides, particularly those residing in underrepresented or unexplored regions. Strategies to enhance this exploration often involve generative and evolutionary algorithms designed to produce peptide libraries with broad structural and functional diversity. For example, Capecchi *et al.* proposed using genetic algorithms to populate peptide space by generating over one million unique sequences, revealing that evolutionary computation can effectively sample distant regions of sequence space that are inaccessible through traditional design methods.^17^ In addition, sequence-based deep generative models have emerged as powerful tools for learning complex peptide sequence patterns while ensuring the generation of novel candidates with diverse scaffolds and biological potential.^173^ These approaches collectively contribute to a more comprehensive exploration of peptide chemical space, supporting the discovery of functionally rich and previously overlooked molecules.

## 5 Perspectives and outlook on peptide design

One of the major challenges in peptide design (Table 3) is the limited availability of standardized and curated datasets encompassing both canonical (cAAs) and non-canonical amino acids (ncAAs). Existing repositories often lack comprehensive structural, physicochemical, and bioactivity data, which restricts the exploration of peptides beyond conventional pharmacological applications. To address this gap, the development of open-access, high-quality peptide databases is critical. Such repositories should integrate data from diverse contexts, including nanomaterials, diagnostics, and biosensors, thereby enabling broader applications and more informed peptide design strategies. Additionally, the necessity to develop more efficient metrics and approaches which allow the systematic study of peptides continues to be one of the great contemporary challenges in drug design.^174,175^

**Table 3:** Future challenges and opportunities in peptide design

Category	Challenge	Perspectives
Data availability	Lack of standardized and curated peptide datasets containing information about canonical (cAAs) and non-canonical amino acids (ncAAs)	Development of open-access, high-quality peptide repositories integrating structural, physicochemical, and bioactivity data across pharmacological and non-pharmacological contexts *e.g.*, nanomaterials, diagnostics, and biosensors
	Incomplete information on peptide chirality and monomer configuration, including asymmetric centers (*e.g.*, sulfoxides, hydroxyproline)	Implementation of systematic annotation protocols including chiral descriptors *e.g.*, MAP4c fingerprint^176^ to improve the interpretability and predictive accuracy of *in silico* models
Data analysis	The quantity and diversity of available compounds are constantly and rapidly increasing	The development of metrics and tools that enable the massive analysis of peptides will allow the correct identification of complex patterns associated with their biological activity
Chemical space study	Limited exploration of the chemical space derived from ncAAs, post-translational modifications, and backbone alterations	Generation of peptide libraries encompassing modified backbones, peptidomimetics, and macrocycles to discover new folding patterns and biological properties
	Identify representative descriptors of the great chemical, physical, and biological diversity of peptides with cAA and ncAA.	The construction of complementary representations (*e.g.*, based on chemical multiverses) will facilitate the study of peptides from different perspectives^177^
Synthesis of canonical and non-canonical peptides	Synthetic constraints for peptides containing ncAAs or chiral sulfur atoms	Development of automated solid-phase synthesis and biotechnological platforms enabling the precise incorporation of stereochemically defined ncAAs
AI-based modeling approaches	Fragmented use of structure-based or ligand-based predictive models without integration of multimodal data	Coupling of structure- and ligand-based models with deep learning frameworks *e.g.*, graph neural networks, attention-based transformers, to generate consensus and interpretable predictions
	Low interpretability of deep learning representations of peptide activity and folding	Application of explainable AI techniques to uncover key structural determinants driving peptide function and stability
	The activity of a peptide must be explained from a holistic perspective that integrates chemical, physical, and/or biological data	The use of AI-based tools that allow for the correct fusion and interpretation of different types of data will aid in the correct decoding of peptide activity

Another significant hurdle involves incomplete information regarding peptide chirality and monomer configuration, particularly at asymmetric centers such as sulfoxides or hydroxyproline. Systematic annotation protocols incorporating chiral descriptors, for instance through MAP4c fingerprints,^176^ could substantially improve the interpretability and predictive accuracy of *in silico* models. Ensuring detailed stereochemical information is essential for accurately modeling peptide folding, activity, and interactions.

The expansion of peptide chemical space remains a key opportunity, especially through the inclusion of ncAAs, post-translational modifications, and backbone alterations. Generating libraries of peptides with modified backbones, peptidomimetics, and macrocycles could reveal novel folding motifs and unique biological properties, opening new avenues for therapeutic and functional applications.

Synthetic challenges also persist, particularly for peptides containing ncAAs or chiral sulfur atoms, which are often difficult to incorporate with precision. Advancements in automated solid-phase synthesis and biotechnological platforms can facilitate the stereochemically defined incorporation of these building blocks, enabling the efficient production of complex peptide structures.^178^

Finally, the application of AI in peptide design presents both opportunities and challenges. Current predictive models are often incomplete or non-integrative, relying separately on structure-based or ligand-based approaches. Integrating these models within deep learning frameworks, such as graph neural networks or attention-based transformers, could generate consensus predictions with improved interpretability.^179^ Furthermore, employing explainable AI techniques will allow researchers to uncover key structural determinants that govern peptide function, stability, and folding, ultimately enhancing the rational design of bioactive peptides.^180^

## 6 Conclusions

The peptide chemical space represents a vast and continuously expanding landscape shaped by the remarkable structural and functional diversity of peptides. This diversity arises not only from canonical amino acid sequences but also from the incorporation of non-canonical residues and post-translational modifications, which collectively generate an immense array of molecular architectures. Accurately navigating and characterizing the peptide chemical space demands a comprehensive suite of molecular descriptors, ranging from sequence-based and connectivity-driven features to 3D structural and physicochemical representations. The availability and integration of diverse descriptors are essential for designing, analyzing, and predicting peptide behavior across different applications. Furthermore, the growing number and complexity of peptide datasets^145,181^ underscore the critical need for robust chemoinformatics and bioinformatics methodologies, whose synergy enables a deeper understanding of peptide function, structure–property (activity) relationships, and the rational design and generation of novel peptide libraries. Moving forward, the development of unified, open-source frameworks and consensus-driven computational standards will be pivotal in capturing the full extent of peptide chemical space and leveraging it for innovation in therapeutics, materials science, nutrition, cosmetics, and beyond.

## Author contributions

Edgar López-López, Jean Paul Sánchez-Castañeda, Massyel S. Martinez-Cortés: investigation, writing – original draft, visualization, formal analysis, writing – review & editing; Cesar de la Fuente-Nunez: conceptualization, writing – review & editing. José L. Medin-Franco: conceptualization, investigation, resources, writing – review & editing, supervision.

## Conflicts of interest

The authors declare no conflicts of interest.

## Abbreviations

3D-VAIF: Three-dimensional vector of atomic interaction field
AMPs: Antimicrobial peptides
anti-MRSA: Methicilin-resistant *Staphylococcus aureus*
BAPs: Bioactive peptides
cAAs: Canonical amino acids
ECFP: Extended connectivity fingerprints
FDA: Food and drug administration
GalNAc: *N*-Acetilgalactosamine
GLP-1 RA: Glucagon-like peptide-1 receptor agonist
HELM: Hierarchical editing language for macromolecules
HNP-1: Human neutrophil peptide 1
MAP: MinHashed atom-pair fingerprint
MXFP: Macromolecule extended atom-pair fingerprint
ncAAs: Non-canonical amino acids
npAAs: Non-proteinogenic amino acids
nsAAs: Non-standard amino acids
PDB: Protein data bank
PLN: Protein line notation
PTMs: Post-translational modification
QED: Quantitative estimate of drug-likeness
SCSR: Self-contained sequence representation
siRNA: Small interfering RNA
SPPS: Solid-phase peptide synthesis
unAAs: Unnatural amino acids

## Data Availability

Data sets are available in GitHub server at: https://github.com/EdgL2/PepChemSpace.
